# The Relationship between Body Appreciation and Self-Esteem and Associated Factors among Omani University Students: An Online Cross-Sectional Survey

**DOI:** 10.1155/2021/5523184

**Published:** 2021-06-23

**Authors:** Atika Khalaf, Iman Al Hashmi, Omar Al Omari

**Affiliations:** ^1^Faculty of Health Sciences, Kristianstad University, Kristianstad, Sweden; ^2^College of Nursing, Sultan Qaboos University, Muscat, Oman; ^3^School of Nursing, Midwifery and Paramedicine, Curtin University, Perth, Western Australia, Australia

## Abstract

**Background:**

Given the rapid pace of globalization and the fact that the Sultanate of Oman is experiencing a significant impact of social media on specifying appearance norms among youth in the country, research into positive body images and self-esteem among young individuals has become a national priority. Whilst body image has been well studied across cultures, both positive body image and the relationship between positive body image and self-esteem among Omani youth have been neglected. The aim of the study was to investigate the relationship between positive body image and self-esteem and associated sociodemographic factors among Omani university students based on gender.

**Methods:**

This cross-sectional study used an online survey consisting of the two questionnaires that are Body Appreciation Scale-2 and Rosenberg's Self-Esteem Scale. A total of 237 students were recruited from Sultan Qaboos University's different colleges.

**Results:**

The results indicated that positive body image has a significant relationship with an individual's self-esteem (*β* = 0.122, *t* = 2.197, *p*=0.038), Cumulative Grade Point Average (cGPA) (*β* = 0.140, *t* = 2.306, *p*=0.022), body mass index (BMI) (*β* = −0.414, *t* = −6.930, *p* < 0.001), monthly household income (*β* = −0.129, *t* = 2.467, *p*=0.029), and the number of social media accounts (≥2, *β* = −0.132, *t* = −2.232, *p*=0.027). In addition, an individual's self-esteem was significantly associated with an individual's cGPA (*β* = 0.231, *t* = 3.592, *p* < 0.001) and mothers' educational level (*β* = −0.130, *t* = −2.065, *p*=0.040) besides body appreciation (*β* = 0.160, *t* = 2.491, *p*=0.013).

**Conclusions:**

The findings of this study shed light on the current status of positive body image among university students of Oman. In light of the new knowledge, we propose health interventions that include strategies such as involvement of family, to maintain and/or promote positive body image perceptions among young individuals and subsequently promote healthy appreciation of the physical appearance and self-esteem.

## 1. Introduction

The body image research has long been dominated by focus on the negative aspects of it including its impact on physical and psychological health [1]. The existing evidence-based knowledge is a valuable source in body image research, but it still lacks an essential part, namely, the focus on the positive body image (PBI) component. The PBI is defined as absolute acceptance of the own body [2]. Since PBI is not only the opposite of negative body image [3], it is important to include the PBI component in order to develop an understanding of the whole body image concept [4]. Further, since body image discrepancies are a growing public health problem leading to psychopathological and behavioral disorders such as body shame, appearance anxiety, depression, and disordered eating [5], an empirically based approach to researching and counteracting body image and its relation to self-esteem is critical.

Self-esteem can briefly be described as a personality trait that is related to emotional and mental well-being [6]. Self-esteem has also been conceptualized as an ecological complex model with the dimensions of global self-esteem, physical self-worth, physical condition, sport competence, physical strength, and attractive body [6]. The valuing of the self is referred to as either high or low, which in reality refers to positive or negative self-esteem [7]. Especially during adolescence, appearance becomes of particular importance for self-esteem, as individuals are ranked more than in any other phase of life. Thus, the physical body plays a very important role in whether an individual is accepted by other individuals. Not being accepted in social contexts can cause doubts about one's appearance and oneself, which can have a devastating effect on both body perception and self-esteem. Thus, body perception and self-esteem are both psychological factors that take shape in social contexts during adolescence [8].

Moreover, the previous literature suggests an association between youths and the social (e.g. family-related conditions such as the number of siblings, obesity, and level of education of the parents), economic (e.g., monthly income, type of housing, and cars in the household) [9, 10], demographic (e.g., age and BMI), and educational factors (academic performance) [11]. Simultaneously, obesity is reported to have negative impacts on one's perception of body image, which is a key element of the development of self-esteem among adolescents [12–14]. Sultanate of Oman is one of the high-income countries that showed a recent rise in the obesity level among adolescents and young adults [15]. In a study directed to college students in Oman, 26.7% and 1.49% of the participants were found to be overweight and obese, respectively [15]. In Oman, the rapid pace in globalization during the last 30 years and the rapid changes in the sociocultural and economic interdependence are probably the contributing factors for the emergence of adolescent obesity [16]. This globalization was accompanied by an increased sedentary lifestyle, changes in dietary practices, easy access to fast food, and decreased level of physical activity [17]. Furthermore, a significant difference between adolescents from different perspectives toward body image was reported [18]. The same results were supported by another research [19].

Although Arab cultures appreciate a fuller body among females as a symbol of good health and wealth, lately in Oman, the global media and the emergence of social media models played an important role in specifying beauty and appearance norms among young adults in the country. A recent study conducted among university students in the United Arab Emirates identified gender differences in the perception of body image where significantly more females desired a thinner body while males desired a heavier body [20]. However, a shift in gender disparities of body image dissatisfaction has been observed in the region where young men showed higher levels of body image dissatisfaction than women [21]. It is therefore important to study this pattern in the Omani context and investigate if this shift in body image perception is a new persistent trend. Assessment of PBI and its relation with self-esteem levels have been conducted among the non-Hispanic white, Hispanics, and Asians [22, 23], yet little is known about Omani adolescents' perception of PBI. Therefore, this study addresses gaps in the literature for PBI among Omani adolescents. Future planning of the study results will help in designing a health intervention to cultivate the PBI among Omani adolescents and young adults to prevent them from suffering the negative psychological effects.

The need for the development of effective strategies to combat the prevailing psychological ill-health in young adults in relation to body image concerns cannot be overemphasized. At the same time, it is paramount to find innovative, sustainable, and culturally sensitive solutions to promote healthy behaviors, attitudes, beliefs, and practices in regard to body image and physical appearance. To reach these goals, we need to explore the landscape of body image perception in Omani populations, and this is to our knowledge the first study attempting to link PBI with self-esteem among Omani healthy individuals in higher education. Therefore, the targeted population in this study will be Omani university students. With established sparse research on a positive experience of the body and self-esteem, it is therefore important that research on the phenomena is conducted to create theoretical and empirical foundations for performing effective health promotion work. Furthermore, exploring the issue will add to the current body of knowledge and will help decision-makers in establishing a new intervention to help youths. PBI has mostly been operationalized as body appreciation (since the development of the Body Appreciation Scale [24, 25]); therefore, the concept of PBI and body appreciation are going to be used as synonyms in the current study. The purpose of this study is, therefore, to investigate the relationship between positive body image and self-esteem and associated sociodemographic factors among Omani university students based on gender.

## 2. Materials and Methods

### 2.1. Design

A cross-sectional study design was utilized to explore the relationship between positive body image and self-esteem based on a gender perspective. The inclusion criteria were [1] being an active student registered in Sultan Qaboos University (SQU), thus having a SQU email address, and [2] being able to read and write in the English language. Students who were registered in postgraduate studies were not invited to participate in the study.

### 2.2. Sample and Setting

This study was conducted at SQU which is the only governmental higher education institution in Oman and it has nine scientific and humanitarian colleges. Students from all over Oman are selected to be enrolled at SQU based on their performance in high school examinations. Top performance students are finally enrolled in the University. Currently, almost 17000 students are registered in undergraduate and postgraduate studies. The sample who met the inclusion criteria and voluntarily participated in the study was 237 male and female university students, aged between 18 and 35 years.

### 2.3. Instruments

#### 2.3.1. Demographics

The participants answered sociodemographic questions including gender, age, marital status, Cumulative Grade Point Average (cGPA), sleeping hours, time spent on social media, number of social media accounts, physical meetings with friends, parents' occupational status and level of education, and number of siblings, as well as their heights and weights.

#### 2.3.2. Body Appreciation

Participants also answered the Body Appreciation Scale-2 (BAS-2) [26] which is a 10-item scale measuring positive body image. All items are rated on a 5-point scale, ranging from 1 (*Never*) to 5 (*Always*). The BAS-2 is composed of one dimension with good internal reliability (Cronbach *α* = 0.91–0.94) and stability (*r* = 0.90) [27]. BAS-2 aims to gain an understanding of body appreciation from different perspectives: acceptance of the body regardless of weight, shape, and deficiencies, respect for the body's needs and commitment to healthy behaviors, positive thoughts about the body regardless of physical appearance, and awareness of unrealistic body ideals. The scale of the questionnaire ranges from 10 to 50 points, with a midpoint of 30 points. This means that all scores from 30 and above indicate a high body appreciation [25]. Permission to use the survey was obtained from the owner of the instrument (Professor Tracy Tylka) prior to the data collection.

#### 2.3.3. Self-Esteem

Participants completed the Rosenberg Self-Esteem Scale (RSES; Rosenberg, 1965) which is a 10-item 4-point scale, ranging from 1 (*strongly agree*) to 4 (*strongly disagree*), which measures global self-worth by measuring both positive and negative feelings about the self. The RSES scale is extensively used in cross-cultural studies in up to 53 different nations and is composed of one dimension with good internal reliability (Cronbach *α* = 0.81) and stability [*r* = 0.82] [28]. The total score is 40 and higher scores indicate higher self-esteem; however, a score less than 15 may indicate problematic low self-esteem. According to the Department of Sociology, Maryland University, “The Rosenberg Self-Esteem Scale may be used without explicit permission.” [29]. In the scoring of the total, five of the items here were reversed and needed to be entered as such in the dataset.

### 2.4. Procedures

This study was initiated by conducting an ethical approval from the research ethics committee at the College of Nursing, Sultan Qaboos University (SQU) (CON/NF/2020/17). After the research was approved, the researchers uploaded the research survey which was composed of Body Appreciation Scale-2 (BAS-2), Rosenberg's Self-Esteem Scale (RSES), and some demographic variables to Google Forms and sent it through public relation email account at SQU to all active undergraduate students at SQU. The email included an information sheet which stressed the free nature of participation in the study. It also emphasized the anonymity of the participants. The information letter was followed by a consent statement and a link that lead the participants to the online survey.

### 2.5. Ethical Considerations

The Helsinki Declarations' ethical recommendations and principles for conducting scientific work were followed during the planning and implementation of the project. Information letter and consent were sent by email. By pressing a link stating “I have read and understood the information and consent to participate in the study,” the participants were directed to the survey page. Contact information to the principal researcher was provided in case the participants had any questions related to the planned study or the procedures for the data collection. Furthermore, the questionnaires were created in Google Forms which is a secure web application for building and managing online surveys. Although no personal data were collected, the results were reported on a statistical group level.

### 2.6. Data Analyses

Data was entered to SPSS version 26. Although no internal dropouts were registered, an operationalizing of the data initiated all analyses. The operationalizing phase included the following: (1) self-reported anthropometric variables were used to calculate the participants' body mass index (BMI), (2) categories with low frequencies were merged (e.g., parents with master's or higher level of education were merged with those having university education), and (3) creation of dummy variables from the categorical variables for the linear regression analyses.

To describe the sample, means, standard deviations (SD), and frequencies were used. In the next step, gender differences in body appreciation, self-esteem, and sociodemographic factors were tested using the chi-square test and independent *t*-test. Finally, using linear regression analysis (backward entry), we examined the association between body appreciation (dependent variable) based on the total score of BAS-2 and the self-esteem scores of RSES (dependent variable) as well as the sociodemographic factors (independent variables). The level of significance was set at *p* < 0.05 for all tests.

## 3. Results

A total of 237 students responded to the online survey. The majority of them were females (62.7%) and unmarried (92%) and had a mean age of 22.0 (SD = 2.2). There were no significant differences when comparing males with females except for the cGPA (*p*=0.013). The majority of the parents had a secondary school education or less (76.3% and 85.2% for fathers and mothers, resp.) and at least one of them had an occupation (51.5%). Most of the students had a household income that was 1500 Omani Riyals or less (79.8%) (Table 1).

Furthermore, almost half (49.6%) of the students have at least two social media accounts and 50.3% had one account. Mean time spent on social media was 4.7 hours ± 2.3 and mean sleeping time was 7.4 ± 1.7 hours with no significant difference between males and females. Many students reported that they do not have weekly physical meetings with friends (46.0%) and 27.0% met their friends once a week; the rest of them (27.0%) met their friends two times or more a week. In addition, the mean body appreciation score was 38.6 ± 8.5 and self-esteem 28.2 + 3.2 with no significant gender difference (Table 1).

This study was conducted to determine if sociodemographic factors are associated with individuals' likelihood to appreciate their bodies and increase their self-esteem. It was hypothesized that the BMI, age, gender, and higher levels of income and education as well as the presence of siblings to support the individual choices in life and confirm the identity of the body image will positively associate with body appreciation and self-esteem. To test this hypothesis, multiple linear regression analysis was used.

Results showed that 21.6% of the variance in the body appreciation is accounted for by the included variables (Table 2), collectively [*F* (5, 231) = 12.76, *p* < 0.001]. Looking at the unique individual contributions of the variables (Table 2), the result shows that BMI value (*β* = −0.414, *t* = −6.930, *p* < 0.001) is negatively associated with body appreciation score, while self-esteem score (*β* = 0.122, *t* = −2.197, *p* = 0.038) is positively associated with body appreciation score. Furthermore, the result also revealed that students who have a monthly household income >1500 OMR (*β* = −0.129, *t* = 2.467, *p*=0.029) and students with two or more social media accounts (*β* = −0.132, *t* = −2.232, *p*=0.027) are less likely to appreciate their body. On the contrary, students with cGPA between 2 and 2.9 (*β* = 0.140, *t* = 2.306, *p*=0.022) are more likely to appreciate their body (Table 2).

For the self-esteem based on RSES, the result showed that 7.7% of the variance in the self-esteem is accounted for by the included variables (Table 3), collectively {*F* (3, 233) = 6.476, *p* < 0.001}. The result showed that body appreciation score (*β* = 0.160, *t* = 2.491, *p*=0.013) is positively associated with self-esteem score. Similarly, those with cGPA less than 2 (*β* = 0.231, *t* = 3.592, *p* < 0.001) are more likely to have higher self-esteem, while students who have a mother with secondary school education (*β* = −0.130, *t* = −2.065, *p*=0.040) are less likely to have high self-esteem (Table 3).

This suggests that body appreciation and self-esteem are intercorrelated. Furthermore, the students' cGPA is shown to associate with both body appreciation and self-esteem in a positive way.

## 4. Discussion

The aim of the current study was to explore the relationship between PBI and self-esteem among Omani university students. Our result showed that BMI value and having more than one social media account are negatively associated with body appreciation scores, while body appreciation score is positively associated with higher levels of self-esteem.

In the current study, higher body appreciation was positively associated with higher levels of self-esteem, which is consistent with previous research findings [30–32]. People who perceive themselves as disfigured and are unable to view themselves in a mirror have negative body image [33]. They complain of several negative physical and psychosocial experiences like low self-esteem, low self-control [33–35], and depression [36, 37]. On the opposite side, people who have positive body image are accepting and acknowledging the individuality and functionality of their own bodies [26], which lead them to accept and appreciate their bodies unconditionally as they focus more on what their body can do than on how their body looks [38]. The positive thoughts about the body that individuals develop may lead them to be more satisfied with their lives and have a high level of self-esteem [30–32]. Therefore, educational settings, including universities, should pay attention and show initiation to develop strategies to enhance students' positive body image and subsequently their self-esteem such as providing educational courses and conducting inspirational campaigns.

Opposite to self-esteem, BMI was negatively associated with PBI in the current study. It seems BMI stayed one of the most significantly associated factors with body image. In a study conducted by Keshk et al. [31], the researchers administered figures scale to examine the preferred body size from university students' perspective. Most participants preferred photos of people who are thin. This is in line with several previous studies [39–41]. Contrary to the current study finding, in a Kenyan study, participants preferred “overweight or obese” body size [42]. Another study examined the body size preference among university female students in five different countries. A significant difference in the participants' perception of the preferred body size was noticed [43]. Similarly, a study conducted in another Gulf country found that university students had a clear discrepancy between their actual, perceived, and ideal body image [9]. This is mostly explained due to the impact of the global media and the emergence of social medial models who played a role in changing the perception of body beauty among young Omani generations. In the past, Arab culture preferred a fuller body size over a thinner size and they believed it was a sign of good health. In the current study, it was evident that positive body image negatively correlated with the number of social accounts the individual has. Sociocultural factors play a significant role in explaining the variation between current and previous studies' findings. Therefore, there is a need for more studies to explore the body image and its relation to the accurate body weight, perception of the body and its functionality, and possible body image concerns within the Omani context. In addition, there is a need for longitudinal studies to explore the predictors rather than the associated factors. Based on the identified predictors, healthcare professionals can create interventional programs to negate the predictors which may negatively impact the body image as well as those that might promote the PBI perception in Omani youths.

The relation between body appreciation and self-esteem can be explained by the current role of mass and social media in promoting thin body beauty. In the current study, students who have more than one social media account are less likely to appreciate their bodies. University students who have more than one account are most likely encountering proeating disorder groups who promote their ideas through social media [44]. These groups promote thin body beauty as a perfect body and mass media produce the same pressure in public as it is always promoting thin body beauty ideal [45, 46], which put pressure on the public and might lead to negative ideas and negative attitudes toward obese and overweight individuals. University students need to be provided with social media literacy training, increase their critical thinking about social media content and messages, and limit their engagement in harmful practices recommended by social media influencers. There is also a need to create interventional programs using social media to counteract the current wave of thin body idealization in social media.

Several other unique variables come to play an important role in being associated with PBI. Students who belong to families with high income do not appreciate their body image. The association can be indirectly explained. Those students have the financial capacity to pay for restaurants; hence, they might consume more fast foods which has been proven to be related to increased risk for obesity. Obesity in turn has negative impacts on one's perception of positive body image, which is a key element of the development of self-esteem among adolescents [12–14]. This might explain the current study findings. Another interesting association in the current study is the association between mothers' level of education and positive body image. Adolescents and youths who have mothers with low educational level do not appreciate their body appearance. Although previous studies such as Khalaf et al. [9] showed that fathers' level of education was associated with body image discrepancies in young female university students; mothers seem to play a similarly significant role in the cohort of the current study. In addition, mothers with a low level of education may not have enough knowledge or the required tools to support and educate their children about the different aspects of body image, physical appearance, and body functionality, which might lead to the development of body image concerns in adult age. There is, therefore, a need for family interventional programs in which the family as a unit is included to magnify the positive impact of the program on university students. Educational sessions for prenatal mothers about PBI are warranted as well to prepare them for their parental role which includes enhancing the perception of positive body image among their future children.

This study did not come without limitations, the convenient sampling technique limits the study generalisability though the participants are heterogeneous and belong to different Omani governates. Yet, the proportion of females was higher than males and this might have impacted the significant differences between the genders. The nature of the study design limits the temporal relationship between the study variables, thus more longitudinal studies are recommended in the future. In addition, possible self-recalling bias cannot be avoided in this type of studies. Thus, more research replicating this study with the use of different methodological approaches may add to the current study's findings.

## 5. Conclusions

This study aimed at investigating the relationship between PBI and self-esteem and associated factors among Omani university students based on gender. We found a significant positive association between PBI and self-esteem with no significant difference between female and male students. The results contributed to the body of existing knowledge and a better understanding of PBI from Omani culture. By increasing knowledge and awareness about body image aspects among Omani university students, the study might promote broader health and social dialogue about the need to create an arena for debate and discussion of the youth's wellbeing from the public health perspective. Thus, faculty members, policymakers, and social media influencers need to highlight and integrate the impact of body image concerns on the health and wellbeing of the youth population in future plans.

## Figures and Tables

**Table 1:** Characteristics of the participants based on gender and presented in column percentages, *n* (%), and mean (standard deviation).

Variable	Female *n* = 149	Male *n* = 88	Total *n* = 237	*p* value^*∗*^
	*n* (%)			
*cGPA*				0.013
Less than 2	5 (3.4)	2 (2.3)	7 (3.0)	
2–2.9	91 (61.1)	70 (79.5)	161 (67.9)	
3–4	53 (35.6)	16 (18.2)	69 (29.1)	

*Marital status*				0.130
Unmarried	134 (89.9)	84 (95.5)	218 (92.0)	
Married	15 (10.1)	4 (4.5)	19 (8.0)	

*Father's educational level*				0.563
Less than primary	35 (23.5)	25 (28.4)	60 (25.3)	
Higher primary	30 (20.1)	12 (13.6)	42 (17.7)	
Secondary school	48 (32.2)	31 (35.2)	79 (33.3)	
University	36 (24.2)	20 (22.7)	56 (23.6)	

*Mother's educational level*				0.935
Less than primary	50 (33.6)	33 (37.5)	83 (35.0)	
Higher primary	40 (26.8)	23 (26.1)	63 (26.6)	
Secondary school	36 (24.2)	20 (22.7)	56 (23.6)	
University	23 (15.4)	12 (13.6)	35 (14.8)	

*Parents' occupation status*				0.868
Only one is working	78 (52.3)	44 (50.0)	122 (51.5)	
Both are working	17 (11.4)	12 (13.6)	29 (12.2)	
None of them is working	54 (36.2)	32 (36.4)	86 (36.3)	

*Household income, OMR* ^*#*^				0.573
Less than 500	40 (26.8)	20 (22.7)	60 (25.3)	
501–1000	56 (37.6)	29 (33.0)	85 (35.9)	
1001–1500	23 (15.4)	21 (23.9)	44 (18.6)	
1501–2000	12 (8.1)	8 (9.1)	20 (8.4)	
More than 2000	18 (12.1)	10 (11.4)	28 (11.8)	

*Number of accounts on social media*				0.986
One account	75 (50.3)	45 (51.1)	120 (50.6)	
Two-three accounts	37 (24.8)	22 (25.0)	59 (24.9)	
Four or more accounts	37 (24.8)	21 (23.9)	58 (24.5)	

*Physical meetings with friends/week*				0.284
Do not meet anyone	72 (48.3)	37 (42.0)	109 (46.0)	
Once a week	42 (28.2)	22 (25.0)	64 (27.0)	
Twice or more a week	35 (23.5)	29 (33.0)	64 (27.0)	
	m (SD)			
Age	21.9 (2.3)	22.2 (2.0)	22.0 (2.2)	0.372
BMI	23.2 (5.8)	23.1 (4.9)	23.2 (5.5)	0.869
Body appreciation (BAS-2)^1^	39.2 (8.5)	37.7 (8.3)	38.6 (8.5)	0.174
Self-esteem (RSES)^2^	28.4 (3.3)	27.9 (3.1)	28.2 (3.2)	0.192
Time spent on social media (hours)	5.0 (2.3)	4.3 (2.2)	4.7 (2.3)	0.042
Sleeping hours	7.4 (1.8)	7.4 (1.6)	7.4 (1.7)	0.900
Number of siblings	7.9 (3.8)	7.8 (3.4)	7.9 (3.6)	0.825

^*∗*^ *p* value based on chi-square (categorical variables) and *t*-test (comparing means of continuous variables). BAS-2 = Body Appreciation Scale-2; RSES = Rosenberg Self-Esteem Scale. ^#^OMR = Omani riyals. ^1^The total score is 50; higher scores indicate higher appreciation of the body. ^2^The total score is 40; higher scores indicate higher self-esteem.

**Table 2:** Association between body appreciation and sociodemographic factors as well as self-esteem.

	*B*	SE	*β*	*t*	*p*
(Constant)	44.066	5.331		8.266	<0.001
BMI	−0.642	0.093	−0.414	−6.930	<0.001
RSES score	0.322	0.154	0.122	2.084	0.038
Household income, >1500 OMR	−2.705	1.231	−0.129	−2.197	0.029
Social media, ≥2 accounts	−2.235	1.001	−0.132	−2.232	0.027
cGPA, 2–2.9	2.528	1.096	0.140	2.306	0.022

Only significant variables are presented. BMI = body mass index, RSES = Rosenberg Self-Esteem Scale, OMR = Omani riyal.

**Table 3:** Association between self-esteem and sociodemographic factors as well as body appreciation.

	*B*	SE	*β*	*t*	*p*
(Constant)	25.971	0.971		26.752	<0.001
BAS-2 score	0.061	0.024	0.160	2.491	0.013
cGPA, <2	4.365	1.215	0.231	3.592	<0.001
Mother's level of education, secondary school	−0.983	0.476	−0.130	−2.065	0.040

Only significant variables are presented. BAS-2 = Body Appreciation Scale-2.

## Data Availability

The datasets generated and/or analyzed during the current study are available from the corresponding author on reasonable request.
